# Mechanical Assessment and Hyperelastic Modeling of Polyurethanes for the Early Stages of Vascular Graft Design

**DOI:** 10.3390/ma13214973

**Published:** 2020-11-05

**Authors:** Said Arévalo-Alquichire, Carlos Dominguez-paz, Manuel F. Valero

**Affiliations:** 1 Energy, Materials and Environmental Group, GEMA, Faculty of Engineering, Universidad de La Sabana, Chía 140013, Colombia; saidaral@unisabana.edu.co (S.A.-A.); carlos.dominguez2@unisabana.edu.co (C.D.-p.); 2 The Doctoral Program of Biosciences, Universidad de La Sabana, Chía 140013, Cundinamarca, Colombia; 3 Department of Prototypes and Manufacturing, Faculty of Engineering, Universidad de La Sabana, Chía 140013, Colombia; 4 Department of Chemical and Biotechnological Processes, Faculty of Engineering, Universidad de La Sabana, Chía 140013, Colombia

**Keywords:** polyurethane, vascular graft, hyperelastic, compliance, biomechanics

## Abstract

The material design of vascular grafts is required for their application in the health sector. The use of polyurethanes (PUs) in vascular grafts intended for application in the body appears to be adequate due to the fact that native tissues have similar properties as PUs. However, the influence of chemical structure on the biomechanics of PUs remains poorly described. The use of constitutive models, together with numerical studies, is a powerful tool for evaluating the mechanical behavior of materials under specific physiological conditions. Therefore, the aim of this study was to assess the mechanical properties of different PU mixtures formed by polycaprolactone diol, polyethylene glycol, and pentaerythritol using uniaxial tensile, strain sweep, and multistep creep-recovery tests. Evaluations of the properties were also recorded after samples had been soaked in phosphate-buffer saline (PBS) to simulate physiological conditions. A hyperelastic model based on the Mooney–Rivlin strain density function was employed to model the performance of PUs under physiological pressure and geometry conditions. The results show that the inclusion of polyethylene glycol enhanced viscous flow, while polycaprolactone diol increased the elastic behavior. Furthermore, tensile tests revealed that hydration had an important effect on the softening phenomenon. Additionally, after the hydration of PUs, the ultimate strength was similar to those reported for other vascular conduits. Lastly, hyperelastic models revealed that the compliance of the PUs showed a cyclic behavior within the tested time and pressure conditions and is affected by the material composition. However, the compliance was not affected by the geometry of the materials. These tests demonstrate that the materials whose compositions are 5–90–5 and 46.3–46.3–7.5 could be employed in the designs of vascular grafts for medical applications since they present the largest value of compliance, ultimate strength, and elongation at break in the range of reported blood vessels, thus indicating their suitability. Moreover, the polyurethanes were revealed to undergo softening after hydration, which could reduce the risk of vascular trauma.

## 1. Introduction

Cardiovascular diseases (CVDs) remain the leading cause of death in high- and middle-income countries. Despite the efforts of governmental and non-governmental organizations to improve people’s diet and lifestyle to prevent such diseases, 31% of deaths worldwide are caused by CVDs, according to the World Health Organization [1]. Stroke and ischemic heart attacks are the top pathologies of this non-communicable diseases [2], which are commonly related to the narrowing of blood vessels. Although drugs are used to prevent vessel failure, a vascular bypass, which is a surgical intervention where a natural, autologous, or synthetic graft is used to replace or detour the blockage vessel, is often used [3] and is believed to be the optimal choice for patients requiring long-term revascularization (with a lifespan of more than two years) [4].

Autologous grafts, such as those utilizing the saphenous vein, are the gold standard in vascular bypass surgery [5] because they have close to exact biomechanical behavior. However, previous vascular surgery and other pathologies are contraindications for the use of autologous grafts [6], as well as high morbidity at the surgery site [7]. Synthetic polymer graft materials such as Dacron and PTFE (polytetrafluoroethylene) are widely used as a solution for vascular replacement [8]. Nevertheless, their compliance values are much lower than that of a native blood vessel. This may lead to a mismatch, causing a certain resistance to any change in diameter while maintaining the constant wall shear stress of natural arteries [9]. Polyurethanes (PUs) have emerged as a solution to the compliance mismatch of Dacron and PTFE. It has been established that PUs have mechanical properties that are similar to those of native tissue, including a low modulus, which favors compliance [10]. Nezarati et al. [9] reported that PUs with a low modulus and high tensile strength could be used for a vascular graft with a high compliance and burst pressure.

In addition to compliance, the viscoelasticity of blood vessels is another biomechanical aspect that has an important role in graft design. This is because vessels convert pulsatile flow from the heart into continuous flow, storing part of the propulsion energy during systole and restoring it to the circulation during diastole [11]. Previous studies have investigated the ability of different replacement grafts to comply with these requirements, including tissue-engineered vascular grafts [12], natural grafts [4], and synthetic grafts [13].

PUs have a segmented structure composed of hard and soft segments, which makes it possible to tailor their physicomechanical properties. PUs are obtained by the reaction of a polyol or blend of polyols with a diisocyanate, where the nonreactive chain of the polyol is the soft segment, providing flexibility, and the hard segment comprises the urethane bond, which grants mechanical strength. Additionally, a chain extender or crosslinker can be used to increase the molecular weight of the hard segment [14]. The chemical structures of polyols and diisocyanate natures have been used to tune the physicochemical and mechanical properties, such as water swelling, thermal stability, elongation at break, ultimate strength, and hardness, as reported in previous works [15,16,17]. In particular, it has been reported that their elastomeric behavior is similar to that of native tissue, and their viscoelasticity has been studied for vascular grafting purposes [18,19]. To the best of our knowledge, information about the time-dependent behaviors of PUs under physiological conditions is still scarce. Despite this, characterization has been carried out in commercial grafts such as Dacron grafts [20].

We have previously reported on the thermomechanical characterization of the polyurethanes studied here [21]. Dynamic–mechanical–thermal analysis revealed a typical elastomeric behavior and the glass transition temperature ranged from −44 to −37 °C. Therefore, a rubber-like behavior was observed, in agreement with previous findings [22].

Therefore, this study considered the mechanical properties of a group of PUs synthesized from a mixture of polycaprolactone diol, polyethylene glycol, and pentaerythritol, and evaluated their potential use in vascular grafts. The polycaprolactone diol and polyethylene glycol were used as the soft segment, while pentaerythritol and isophorone diisocyanate constituted the hard segment. Their static and dynamic behaviors were evaluated by performing uniaxial tensile tests, strain sweep tests, and multistep creep–recovery tests. The elastic behavior was modeled using a nonlinear hyperelastic model. This constitutive model formulation was used to simulate the behavior of the PUs over time as a vascular conduit under physiological conditions. As such, the material was analyzed under sinusoidal pressure ranges at different graft radii and thicknesses. The effect of the PU mixture composition was evaluated to broaden the comprehension of the material’s mechanics and its performance for application in the early stages of vascular graft design, allowing for the selection of materials with the greatest potential to be used in further studies and designs.

Before continuing, the reader should know that the results presented here are limited by the conditions of the test performed. The results for uniaxial load are presented along with modeling results based on incompressible material and further used to identify the advantages of each material and select the most promising candidates for further design and numerical analyses.

## 2. Materials and Methods

### 2.1. Materials

Polyethylene glycol (PEG, Av. Mn ~1000 g/mol) was purchased from Merck KGaA (Darmstadt, Germany). Polycaprolactone diol (PCL, Av. Mn ~2000 g/mol), isophorone diisocyanate (IPDI), and *N*,*N*-dimethylformamide (DMF) were purchased from Sigma-Aldrich (St. Louis, MO, USA). Pentaerythritol (PE) was obtained from Alfa Aesar (Heysham, UK), and phosphate-buffered saline (PBS) was obtained from VWR (Randor, PA, USA). In this study, PEG and PCL played the role of polyols, while PE and IPDI comprised the hard segment. DMF was used as the reaction solvent.

### 2.2. Synthesis of PUs

PUs were synthesized as previously reported [21]. Briefly, PCL and PEG were dissolved in DMF at 70 °C. IPDI was then added to the polyol blend and allowed to react for 15 min at 70 °C. Next, the second solution of PE in DMF was added, and the solvent was evaporated for at least 5 h. Finally, the solution was poured onto a glass surface, and thin films were made with the help of an Elcometer 3580 casting knife film applicator (Elcometer Ltd., Manchester, UK) with a gap of 150 µm. The PU was cured for 12 h at 110 °C. Four blends were synthesized with the compositions listed in Table 1. These blends were selected based on results from our previous work showing they had better phase mixing and damping behavior of those tested [21].

### 2.3. Mechanical Testing

All mechanical tests were performed on a DMA850 dynamic mechanical analyzer (TA instruments, New Castle, DE, USA) modified with a film clamp. All experiments were performed at 37 °C, and the tested rectangular samples all had dimensions of 20 mm length, 2.5 mm width, and 0.3–0.5 mm thickness. Thickness was measured with a digital caliper prior to each test. Five separate samples of each PU were tested. Additionally, for each sample and procedure, an initial preload of 0.001 N and soak time of 5 min at 37 °C were used. The samples were fixed to the clamp with a torque of 0.4 in∙lbf using a QDRIVER3 adjustable torque screwdriver (Snap-on, Kenosha, WI, USA). The strain was measured by the sensors installed in the equipment.

This work evaluated the performance of non-hydrated and hydrated PUs. Each PU was hydrated in 1× PBS for 24 h at 37 °C with gentle agitation.

**Figure 1:**
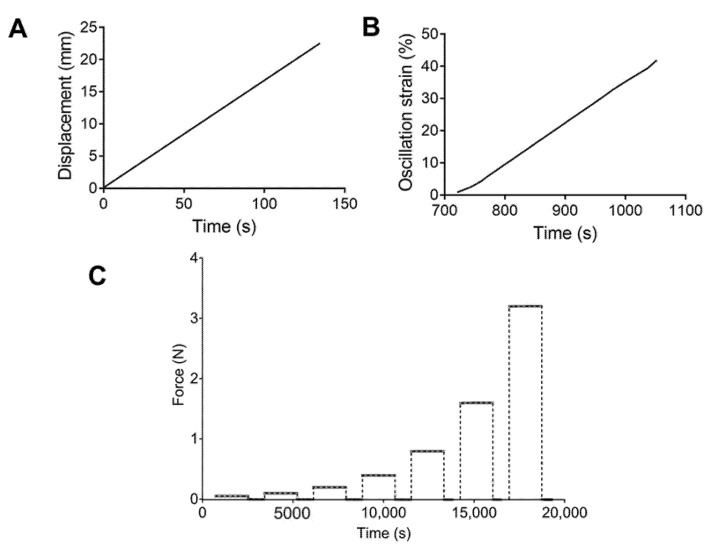
Schematic representation of the (**A**) tensile test, (**B**) strain sweep, and (**C**) cycles of forces applied for the creep test.

For the tensile tests, a rate control program was set up with a strain ramp of 10 mm/min (Figure 1A). Engineering stress–strain curves were obtained, and the ultimate strength and elongation at break were calculated. Strain sweep tests were performed using an oscillation program at a frequency of 1 Hz and a strain range of 1.0–50.0%, with 1.0% increments (Figure 1B). The storage and loss modulus were recorded. Finally, multistep creep–recovery load tests were conducted. Samples were subjected to loads of 0.05, 0.1, 0.2, 0.4, 0.8, 1.6, and 3.2 N. Values of 1800 and 900 s were used for the creep and recovery times. Figure 1C describes the loads applied to each PU.

### 2.4. Hyperelastic Modeling

For the hyperelastic model, a strain energy function W(**C**) of the right Cauchy–Green tensor, defined as **C** = **F**^T^**F**, was chosen so that a constitutive equation could be found, where **F** is the deformation gradient tensor. For the present study, the Mooney–Rivlin model was chosen [23,24,25] such that W= ***f*** (I1, I2,I3). I1, I2, and I3 are the three principal strain invariants of **C** and are defined in terms of the stretch ratios in the principal direction as

(1) $$I_{1}=\lambda_{1}{}^{2}+{\;\lambda }_{2}{}^{2}+\lambda_{3}{}^{2}$$

(2) $$I_{2}=\frac{1}{\lambda_{1}{}^{2}}+\frac{1}{\lambda_{2}{}^{2}}+\frac{1}{\lambda_{3}{}^{2}}$$

(3) $$I_{3\;}=\lambda_{1}^{2}\lambda_{2}^{2}\lambda_{3}^{2}$$

(4) $$W=\;\underset{i}{\sum }\underset{j}{\sum }C_{\mathrm{ij}}{\left(I_{1}-3\right)}^{i}{\left(I_{2}-3\right)}^{j}$$

According to Rivlin [26] and considering the conventional assumption of incompressibility for rubber-like materials (I3 = 1), the energy function takes the form presented in (4). Here, C_ij_ is the material constant obtained through curve fitting with the experimental results. Each of the principal Cauchy stresses can be related to the energy through [27]

(5) $$\sigma_{i\;\left(i=1,2,3\right)}=\lambda_{i\;\left(i=1,2,3\right)}\frac{\partial W}{\partial \lambda_{i\;\left(i=1,2,3\right)}}$$

Based on the suggestion by Kumar and Rao [28], a three-parameter Mooney–Rivlin function was used. Additionally, tests were performed under uniaxial conditions. Therefore, the following is true:

$$\sigma_{2}=\sigma_{3}=0$$

$$\lambda_{1}=\lambda \;\mathrm{and}\;\lambda_{2}=\lambda_{3}=\frac{1}{\sqrt{\lambda }}$$

and substituting (4) into (5) and reducing the stress in the load direction ($\sigma_{1}$) is defined by (6)

(6) $$\sigma_{1}=2C_{10}\left[\lambda^{2}-\frac{1}{\lambda }\right]+2C_{01}\left[\lambda -\frac{1}{\lambda^{2}}\right]+6C_{11}\left[\lambda^{3}-\lambda^{2}-\lambda \;+\frac{1}{\lambda \;}+\frac{1}{\lambda^{2}}-\frac{1}{\lambda^{3}}\right].$$

As previously reported, mechanical models can be used to determine the correlation of compliance with uniaxial test results [29]. Therefore, parameters were estimated from the uniaxial tensile test results. An average stress–strain curve of the five tested samples for each PU was calculated, and nonlinear regression was used to calculate the model parameters. The engineering stress ($\sigma_{\mathrm{Eng}}$) and strain ($\varepsilon_{\mathrm{Eng}}$) were transformed into the true strain and stress, as follows:

(7) $$\frac{\sigma }{\lambda }=\sigma_{\mathrm{Eng}},$$

(8) $$\lambda =1+\varepsilon_{\mathrm{Eng}}$$

### 2.5. Modeling Vascular Grafts under Physiological Conditions

The behaviors of the PU-based vascular grafts were studied under simulated physiological conditions (hydration, temperature, and simulated pressure). A sinusoidal arterial blood pressure model was used [30]:

(9) $$P\left(t\right)=P_{m}\left(1+\epsilon \mathrm{Sin}\left(\omega t\right)\right)$$

where ω is the frequency in hertz, t is the time in seconds, $\epsilon =\frac{P_{s}}{P_{m}}$, $P_{s}$ is the amplitude (10 mmHg) of the sinusoidal pressure, and $P_{m}$ is the mean pressure, which was calculated by (10), as shown in previous reports [12]:

(10) $$P_{m}=\;\frac{1}{3}P_{\max}+\;\frac{2}{3}P_{\min}$$

The maximum ($P_{\max}$) and minimum ($P_{\min}$) pressure were 180 and 40 mmHg respectively, so $P_{m}$ was 86.67 mmHg.

The circumferential stresses ($\sigma_{\theta \theta }$) were estimated based on the thick wall theory for vessels reported elsewhere [12,31].

(11) $$\sigma_{\theta \theta }=\frac{P\left(t\right)r_{0}{}^{2}}{{\left(r_{0}+h\right)}^{2}}\left(1+\frac{{\left(r_{0}+h\right)}^{2}}{r_{0}{}^{2}}\right)$$

Here, $r_{0}$ is the graft inner radius under undeformed conditions, h is the graft thickness, and P(t) is the pressure as a function of time, as previously described.

To determine the change in radius, $\sigma_{\theta \theta }$ was used to calculate the stretch from the hyperelastic model, and the radius was calculated as follows:

(12) $$r\left(t\right)={\;r}_{0}\left(\lambda -1\right)+r_{0}$$

Compliance was calculated as follows:

(13) $$\mathrm{Compliance}=\;\frac{r_{i+1}-r_{i}}{r_{i}\left(P_{i+1}-P_{i}\right)}$$

### 2.6. Statistics and Modeling

Mechanical properties from stress–strain curves were analyzed using analysis of variance (ANOVA) and groups were compared using two different post hoc tests. Sidak’s test was used to compare hydration states while Tukey’s test was employed to compare compositions. Before ANOVA, a Shapiro–Wilks test was performed to evaluate the normality (alpha = 0.05). The root mean square error (RMSE) and Lin’s concordance correlation coefficient (CCC) were used to evaluate the goodness of fit and reliability of the hyperelastic models.

The parameter estimation and mathematic modeling were performed in MATLAB 2019b (MathWorks, Natick, MA, USA). The algorithms used in this work are presented in the Supplementary Materials.

## 3. Results and Discussion

### 3.1. Mechanical Assessment

**Figure 2:**
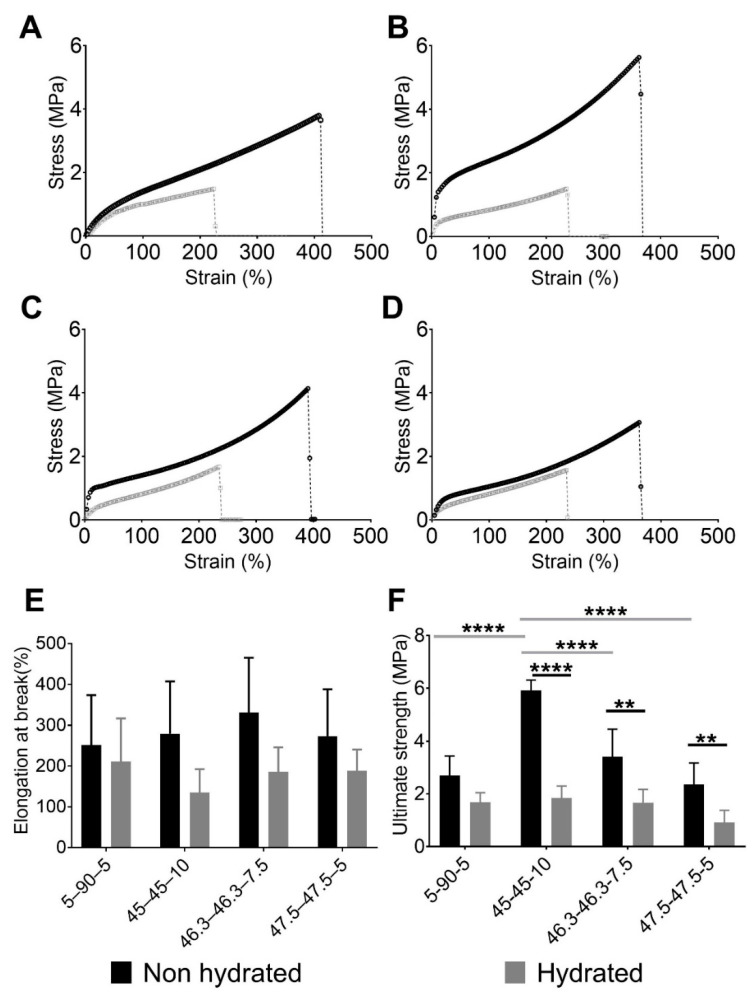
Engineering stress and strain performances of evaluated PUs showing the effect of the composition and softening of PUs after hydration. Stress vs. strain of non-hydrated and hydrated PUs: (**A**) 5–90–5, (**B**) 45–45–10, (**C**) 46.3–46.3–7.5, and (**D**) 47.5–47.5–5. (**E**) Elongation at break values of non-hydrated and hydrated PUs and (**F**) ultimate strengths of non-hydrated and hydrated PUs. In E and F, data are presented as the mean ± standard deviation (n = 5). Gray horizontal bars indicate significant differences between the material compositions, while black horizontal bars show the differences between hydration states. ** 0.001 ≤ *p* < 0.01, and **** *p* ≤ 0.0001.

Figure 2 shows the performances of the PUs in tensile experiments. Figure 2A–D displays the engineering stress and strain curves for each PU composition with their characteristic elastomeric shape. Initially, a small region of elasticity at low deformation was observed, which resulted from hydrogen bonding. Then, the polymer chains uncoiled, producing a moderate stress increase with deformation. Finally, after the strain increased to more than approximately 200%, a “stress-induced crystallization” phenomenon was observed. In that case, the strengthening produced by the chain orientation could ease the formation of new hydrogen bonds [32,33]. This behavior has previously been described for elastomer materials [34,35,36]. In particular, 45–45–10 (Figure 2B) and 46.3–46.3–7.5 (Figure 2C) had the highest stress values. These materials also had the largest amounts of PE, which worked as a crosslinker. In our previous study, 45–45–10 was found to have the largest theoretical hard segment content of 39.22%, while 46.3–46.3–7–5, 47.5–47.5–5 and 5–90–5 had values of 34.09%, 28.36%, and 25.47%, respectively [21]. Therefore, increasing the number of IPDI–PE–IPDI segments, which are hard segments, resulted in more efficient hydrogen bond formation and subsequently increased the reticulation and enhanced mechanical performance [37]. Furthermore, the elastic regions in the stress–strain curves of 45–45–10 (Figure 2B) and 46.3–46.3–7.5 (Figure 2C) were higher than those of 5–90–5 (Figure 2A) and 47.5–47.5–5 (Figure 2D), which had lower PE contents. Hence, fewer IPDI–PE–IPDI segments were available for hydrogen bonding.

The PUs had poorer mechanical performance after being hydrated. The hydrated curves in Figure 2A–D are shorter than the non-hydrated ones. The water inside the PU network acts as a plasticizer, reducing the amount of intermolecular forces like hydrogen bonds [38] and making the material softer and more ductile. This phenomenon was observed for the elongation at break and ultimate strength.

Figure 2E,F show the elongation at break and ultimate strength respectively, of each PU studied. The ultimate strength and elongation at break were evaluated using normality tests and both variables passed (alpha = 0.05). Regarding elongation at break, no significant differences were observed between the PU compositions and hydration states. In terms of the ultimate strength, an effect of the composition was observed in the non-hydrated state. The largest concentration of PE produced PUs with the greatest values, with 45–45–10 reaching an average value of 5.92 MPa. By contrast, the PUs with the lowest concentrations of PE, namely 5–90–5 and 47.5–47.5–5, had stresses of 2.69 and 2.36 MPa, respectively. Statistical analyses supported these findings, with significant differences in ultimate strength found between 45–45–10 and the other PUs. Moreover, the differences were greater between 45–45–10 and the PUs with the lowest concentrations of PE, i.e., 5–90–5 and 47.5–47.5–5 (*p* < 0.01). A comparison of non-hydrated and hydrated states showed that there were reductions in the ultimate strength for all materials after hydration. Significant differences were observed for each pair of hydrated and non-hydrated PUs, except for 5–90–5. However, the ultimate strength values were similar across all of the compositions, with non-significant differences. As described in our previous work [21], 5–90–5 was the most hydrophobic polymer because it is composed of 90% PCL, a hydrophobic polyol. Therefore, it swelled with the lowest amount of water, reducing the mechanical loss.

The values for ultimate strength of the PUs presented in this paper are comparable to those synthesized by Puszka and Kultys [39], who characterized polyurethanes synthetized with poly(oxytetramethylene)diol and poly(hexamethylene carbonate)diol. PUs with a hard segment content of around 30% had an ultimate strength that ranged between 1.90 and 7.50 MPa, while ultimate strength values for our PUs ranged between 2.3 and 5.9 MPa.

The values of the ultimate strength and elongation at break were similar to those in previous reports on natural blood vessels. Coronary arteries, saphenous veins, and internal thoracic arteries had ultimate strength ranges of 0.5–2, 1.5–4, and 1.5–4 MPa, respectively [40]. The elongation-at-break values for these blood vessels ranged from 40% to 100% [40]. These values agreed with those reported by Karimi et al. [41], who found an ultimate strength of approximately 1.44 MPa and an elongation at break of approximately 54% for healthy human coronary arteries. The values for the ultimate strength and elongation of break of the studied PUs after hydration had ranges of 1.85–0.92 MPa and 211–135%, respectively. Therefore, the ultimate strength and elongation at break of 5–90–5, 45–45–10, and 46.3–46.3–7.5 are in the range reported above. The synthesized PUs had properties similar to those of natural blood vessels. Moreover, the observed softening of PUs after hydration is in agreement with previous reports [42,43]. Such softening, mediated by water absorption, could provide advantages for medical applications [14]. Particularly in the case of cardiovascular devices, it could reduce patient discomfort and the risk of vascular trauma [18].

**Figure 3:**
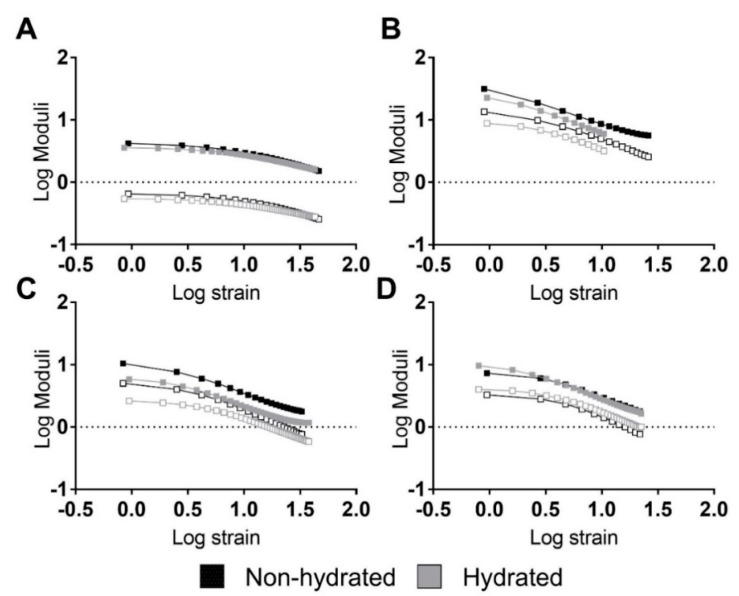
Strain sweeps of the studied PUs revealing strong relations between moduli and strain. Representative performances of hydrated and non-hydrated PUs: (**A**) 5–90–5, (**B**) 45–45–10, (**C**) 46.3–46.3–7.5, and (**D**) 47.5–47.5–5. Filled squares represent the storage modulus while non-filled squares represent the loss modulus.

Strain sweep testing is a tool used to study the viscoelastic behaviors of materials under dynamic conditions [44]. Figure 3 shows the strain sweeps of the previously mentioned materials. Logarithmic moduli and oscillation strain are represented in this figure. The storage ($E^{\prime }$) and loss modulus ($E^{\prime \prime }$) were calculated as follows:

(14) $$E^{\prime }=\;\frac{\sigma }{\varepsilon }\cos\left(\delta \right),$$

(15) $$E^{\prime \prime }=\;\frac{\sigma }{\varepsilon }\sin\left(\delta \right)$$

where σ, ε, and δ are the stress, strain, and phase angle, respectively.

In general, the PUs did not reveal linear viscoelastic regions. Only 5–90–5 (see Figure 3A) exhibited a short linear region at strains lower than 3% and 2% for the non-hydrated and hydrated PUs, respectively. Thus, the PUs had a strong stress and strain dependency, revealing a nonlinear behavior.

A composition effect similar to the one seen in the tensile experiments was identified for the moduli, with higher values for 45–45–10 (Figure 3B), followed by 46.3–46.3–7.5 (Figure 3C), 5–90–5 (Figure 3A), and 47.5–47.5–5 (Figure 3D). Around the hydration state, hydrated PUs displayed similar behavior but had a lower modulus.

**Figure 4:**
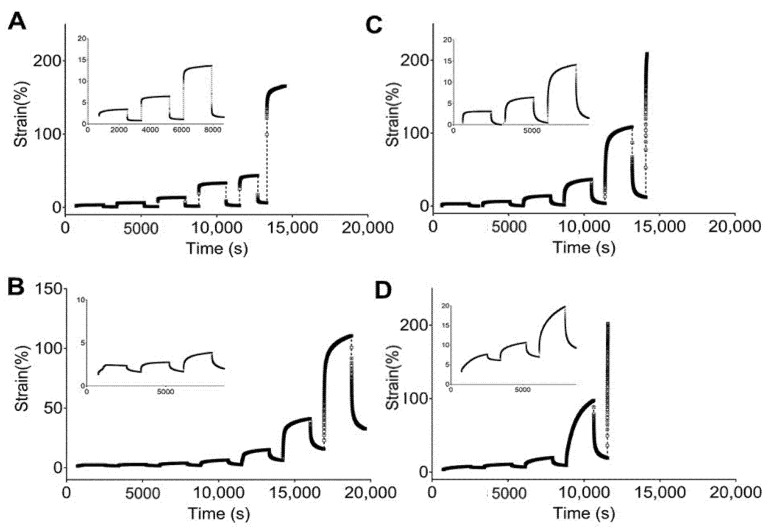
Strain responses over time obtained from creep tests of studied PUs in a non-hydrated state: (**A**) 5–90–5, (**B**) 45–45–10, (**C**) 46.3–46.3–7.5, and (**D**) 47.5–47.5–5.

**Figure 5:**
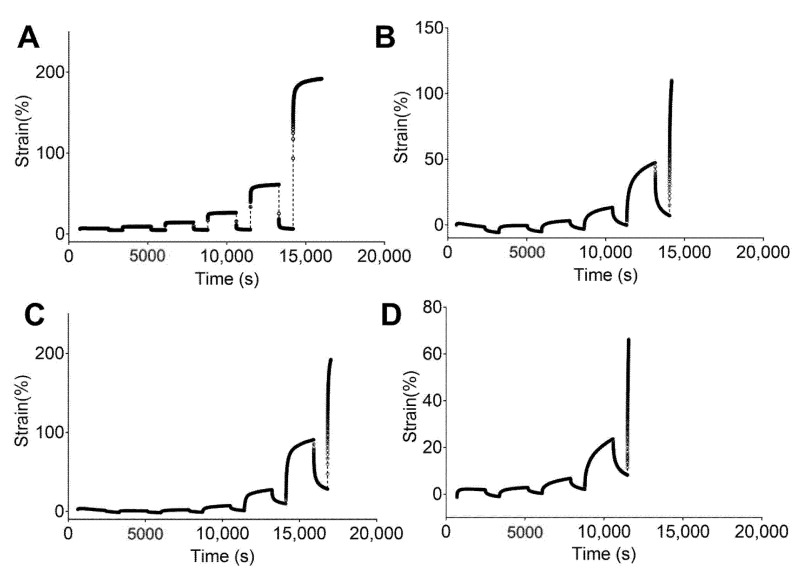
Strain response against time obtained from creep tests for studied PUs in a hydrated state: (**A**) 5–90–6, (**B**) 45–45–10, (**C**) 46.3–46.3–7.5, and (**D**) 47.5–47.5–5.

Figure 4 and Figure 5 show the engineering strain responses obtained from the creep tests of all PUs in the non-hydrated and hydrated states, respectively. In general, the results show that at small loads, there is creep behavior and an elastic response during the recovery, and that the slope increases with the applied load. In particular, Figure 4 shows the creep strain for the non-hydrated state. This figure shows an instantaneous strain response due to the elastic response, followed by viscous flow at the end of the loading stage. During the recovery stage, the strain quickly decreases as a result of the elastic response. Finally, a time-dependent section due to viscous flow is observed that is similar to the original dimensions. However, the deformation became permanent because of the same phenomena.

Figure 4A shows that 5–90–5 has a low viscous flow during the creep time while 47.5–47.5–5 (Figure 4D) has the largest value for this parameter. An instantaneous elastic response is easily observed for 5–90–5, while the rest of the PUs show a greater time dependency. Likewise, permanent deformation was reached at different loads for each polymer, with values of 0.8, 0.2, 0.2, and 0.05 N for 5–90–5 (Figure 4A), 45–45–10 (Figure 4B), 46.3–46.3–7.5 (Figure 4C), and 47.5–47.5–5, respectively. The permanent deformation is important because it could result in a loss of dimensional stability [45].

The PUs in the hydrated state (Figure 5) showed the same behaviors as the non-hydrated PUs, with 5–90–5 (Figure 5A) exhibiting a strong elastic behavior and 47.5–47.5–5 (Figure 5D) displaying the strongest viscous flow during the creep time. Furthermore, 5–90–5 had a strong instantaneous elastic response in the recovery stage. Permanent deformation was reached at 0.4 N for 5–90–5, 0.2 N for 45–45–10 (Figure 5B), 0.1 N for 46.3–46.3–7.5 (Figure 5C), and 0.05 N for 47.5–47.5–5. Water acting as a plasticizer reduced the force required for permanent deformation in cases like 5–90–5 and 46.3–46.3–7.5.

The incorporation of PEG into the matrix enhanced the viscous flow. Therefore, 5–90–5 displayed the greatest elastic behavior while 47.5–47.5–5 had the lowest. Additionally, 47.5–47.5–5 reached permanent deformation at low loads, which could compromise the performance of the vascular graft.

The information presented at this point describes the mechanical behavior of the polyurethanes and the influence of the composition. Moreover, the effect of water uptake was observed for the same mechanical properties. The water swelling reduced the ultimate strength and the elongation at break, and the viscous flow and permanent deformation were reached at lower forces. This points to the relevance of the study of water in the polymeric matrix, which has been poorly described in the literature for vascular graft applications. Materials employed for vascular grafts are placed in contact with physiological fluid and, therefore, swelling could take place, jeopardizing graft performance.

### 3.2. Hyperelastic Modeling

An elastomeric behavior and large extensions were observed in the previously mentioned mechanical assessments of the PUs. The PUs demonstrated a significant strain dependency even at low strain values, and the elastic response and viscous flow were regulated by the PU composition. In this way, the large extension of rubber-like materials and nonlinear elastic behaviors have been expressed using hyperelastic models [46]. Additionally, the hydration of the PUs produced a softening phenomenon. Hence, hyperelastic modeling and further simulations were performed with the hydrated materials, which had behaviors closer to those of an in vivo application.

In the past, PUs have been considered for vascular graft design [9,47] because of their hyperelastic behavior, which can support repeated stress, similar to a native blood vessel [48]. In this way, a hyperelastic model based on the Mooney–Rivlin strain energy density function was used to address the stress and strain behaviors.

The coefficients of the average curves were calculated using three samples of each PU. According to Cook et al., biomechanical models constructed with average values do not produce average results [49]. Hyperelastic models based on averages tend to fail when any coefficient underwent nonlinear change [50], which was the case with the Mooney–Rivlin function used in this work.

A physical interpretation of Mooney–Rivlin parameters was provided by Kumar and Rao [28]. Parameter C_10_, from Equation (5), can be used to calculate the crosslinked density of the polymers following the next equation:

(16) $$v=\frac{C_{10}}{\mathrm{RT}}$$

where R is the universal gas constant and T is the temperature.

The crosslinked density (v) is reported in Table 2, and 45–45–10 showed the largest v. The incorporation of large ratios of PE increased the crosslinking density. The PE used in this work functioned as a chemical crosslinker and, hence, reduction in the PE concentration resulted in reduced crosslinking, and 5–90–5 and 47.5–47.5.5 therefore had the lowest values for crosslinked density.

Likewise, C_01_ represents the deviation from linearity. Large values signify an increase in nonlinearity of the stress–strain curve. 45–45–10 exhibited the largest values, representing the largest deviation from linearity. The additional parameter in the Mooney–Rivlin strain, the C_11_ density function, resulted in the curve having more inflection points [28].

**Figure 6:**
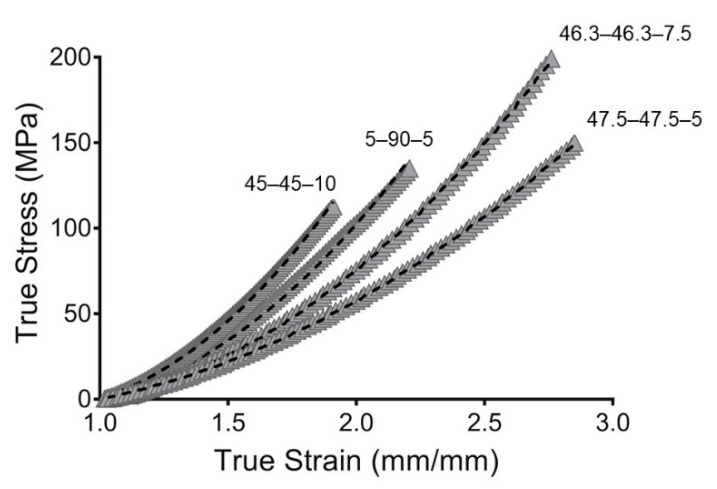
Hyperelastic modeling of true stress and true strain from synthesized PUs. Gray filled symbols represent the mean curve while the dashed line represents the hyperelastic model.

To evaluate the goodness of fit, the root mean square error (RMSE) and concordance correlation coefficient (CCC) were evaluated. The RMSE measures the error between predicted and experimental values. However, the RMSE is scale-dependent, which made it impossible to compare the PU compositions. Likewise, the CCC was used as a reliability index. The CCC is a modification of the Pearson correlation coefficient and is used to assess either how close the data are to the line for the best fit or how far that line is from the 45° line through the origin. This makes it possible to evaluate the correlation and agreement between methods [51]. Additionally, the CCC is scale-independent. The models obtained in this work show great goodness of fit, with value of CCC close to one. This indicated that the models are reliable and represent the behaviors of the PUs well. The fitting is represented in Figure 6.

**Figure 7:**
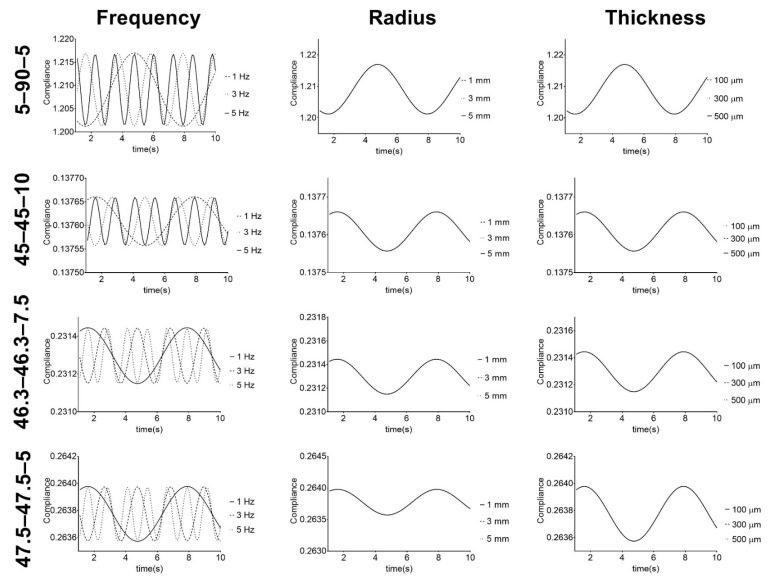
Response of PU-based vascular grafts under simulated physiological mechanical conditions. Compliance given in mmHg^−1^ × 10^4^.

Vascular grafts based on the PUs were studied under simulated physiological mechanical conditions. The frequency, vascular graft inner radius, and thickness were studied in terms of the compliance, and the results are presented in Figure 7.

Figure 7 shows that compliance has a cyclic behavior with pressure frequency variation. The PUs show cyclic variations, but with the same minimum and maximum values for each frequency. These results illustrate the capacity of the PUs to withstand physiological conditions, which is one of the requirements for vascular grafts and is in agreement with the cyclic behavior shown in the work of Valdez-Jasso et al. [52]. Additionally, it shows that for each composition, the radius and thickness of the vascular graft do not affect the compliance behavior. However, there was variation in the compliance values of the graft compositions. These results suggest that for each PU composition, the stiffness of the material does not change under different graft geometries but can be controlled and modified by adjusting the composition. Regarding this, Stewart and Lyman [6] reported that mismatches in both graft compliance and dimensions with the native vessel must be avoided to prevent any possible adverse effect on the performance. Therefore, the obtained results indicate that these PUs, which retained their stiffness when their geometry was changed, are potential materials that could be used to address the compliance mismatch problem for vascular vessels with different diameters and thicknesses.

In terms of the compliance values, PU 45–45–10 had lower values, since it was the stiffest material, while 5–90–5 had the largest values of compliance. The second largest value was recorded for 46.3–46.3–5, but this PU reported the poorest mechanical properties after hydration. 5–90–5 and 46.3–46.3–7.5 stand out because of their good mechanical properties after hydration and compliance close to 45–45–10. This suggests these two PUs can be further used in vascular graft design.

A limitation of the present study was that it did not consider the anisotropy of the vascular vessel, where the heterogeneity of strain affects the stress distribution [53]. This anisotropy is associated with the vessel wall composition, which includes various layers. Recent studies have reported computational models for bilayered grafts with different geometrical constraints and material properties [54]. Characterization of the anisotropic properties of the material requires in situ observations that were beyond the scope of this work, but that could be achieved using the experimental methods mentioned here combined with multiphoton microscopy techniques [55]. Additionally, the second limitation of this paper is the coefficient source. The coefficients presented in this work were only derived from a uniaxial test. Therefore, further studies on different configurations of the mechanical test (e.g., planar and biaxial) should be added to validate the parameters. However, this study aimed to evaluate the hyperelasticity of a group of materials to select the best for vascular graft design. The methods presented in this work can be used to select material for further use in the early stage of vascular graft design.

## 4. Conclusions

Mechanical assessments of the synthesized PUs showed a loss of mechanical properties after hydration, as measured in terms of the ultimate strength and elongation at break. However, softening could reduce the risk of vascular trauma and patient discomfort. The values for the ultimate strength and elongation at break were similar to those reported for coronary arteries and saphenous veins. The non-hydrated and hydrated PUs both showed significant strain dependencies, revealing nonlinear viscoelasticity. Additionally, creep–recovery tests revealed significant elastic behaviors for PUs with large PCL concentrations, while PEG addition enhanced the viscous flow and reduced the elastic performance. Moreover, hydration reduced the elastic region.

The material was modeled using a nonlinear hyperelastic model based on a Mooney–Rivlin strain density function. The RMSE and CCC revealed the good fit and reliability of the experimental data and model. Under simulated physiological conditions, the PUs showed cyclic behaviors, indicating their capacity to sustain physiological conditions. Moreover, the compliance did not change with the radius and thickness, suggesting enhancement of the compliance and vascular geometry mismatch. PUs 5–90–5 and 46.3–46.3–7.5 displayed a better performance after hydration in all tests.

Therefore, the PUs described in this work show the potential to overcome the compliance mismatch problem for vascular vessels with different diameters and thicknesses. Furthermore, the biomechanical modeling presented in this work could be used as a tool in the early design stage for materials and vascular grafts, where the material behavior can be evaluated to predict the best performance for in vivo applications.

Finally, further works should address different loads, such as planar and biaxial loads, to complement the constitutive model and confirm the supposition made about the incompressibility of the material.

## Figures and Tables

**Table 1:** Compositions of the studied polyurethanes (PUs).

Sample Name	PEG (% *w*/*w*)	PCL (% *w*/*w*)	PE (% *w*/*w*)
5–90–5	5	90	5
45–45–10	45	45	10
46.3–46.3–7.5	46.3	46.3	7.5
47.5–47.5–5	47.5	47.5	5

**Table 2:** Hyperelastic model parameters from best regression, goodness of fitness, and reliability parameters.

Sample	C_10_ (MPa)	C_01_ (MPa)	C_11_ (MPa)	V ^a^ (kmol/m^3^)	RMSE ^b^ (-)	CCC ^c^ (-)
5–90–5	5.89 × 10^−1^	−4.16 × 10^−1^	−3.99 × 10^−2^	2.3 × 10^−1^	0.020	0.9996
45–45–10	−1.41	3.03	2.23 × 10^−1^	5.5 × 10^−1^	0.016	0.9999
46.3–46.3–7.5	−8.54 × 10^−1^	1.82	1.32 × 10^−1^	3.3 × 10^−1^	0.006	1.0000
47.5–47.5–5	−8.12 × 10^−1^	1.66	1.01 × 10^−1^	3.2 × 10^−1^	0.028	0.9971

^a^ Crosslinking density, ^b^ root mean square error, and ^c^ Lin’s concordance correlation coefficient.

## Supplementary Materials

The following are available online at https://www.mdpi.com/1996-1944/13/21/4973/s1.
