# Supplementary material for: Mechanical Assessment and Hyperelastic Modeling of Polyurethanes for the Early Stages of Vascular Graft Design
Source: Materials (Basel). 2020 Nov 5;13(21):4973. doi: 10.3390/ma13214973 (PMC7663800; doi:10.3390/ma13214973)
Supplement: Supplementary file 1 [file materials-13-04973-s001.pdf]

materials

Supplementary Materials

Mechanical Assessment and Hyperelastic Modeling of Polyurethanes for the Early Stages of Vascular Graft Design

Said Arévalo-Alquichire, Carlos Dominguez-paz and Manuel F. Valero

Supplementary S1: MATLAB algorithm for average curve calculation

```
%Calculating average curve from three sets of data per PU, the code first call data from txt files and extract strain and stress in 'x' and 'y' variables, respectively. Then, the maximum strain is identified and the minimum between the three sets is used as a limit for interpolation. Interpolated data is saved in a variable called 'out'.
clc;
%Importing data
data1=PU1011H2;
data2=PU1011H3;
data3=PU1011H4;
%Extracting strain in 'x' variables, and stress in 'y' variables
y1=data1.Stress;
x1=data1.Strain;
y2=data2.Stress;
x2=data2.Strain;
y3=data3.Stress;
x3=data3.Strain;
%Chose of interpolation limit
max1=max(x1);
max2=max(x2);
max3=max(x3);
Global=[max1,max2,max3];
minG=min(Global);
%Defining the new 'x' space between 1 and the minimum of maximum strains
x=linspace(1,minG, 100);
%Interpolating the new 'x' vector in each set of data
yy1=interp1(x1, y1, x);
yy2=interp1(x2, y2, x);
yy3=interp1(x3, y3, x);
%Average of the three interpolating values
y=mean([yy1; yy2; yy3], 1);
%Saving the results
out=[x',y'];
```

Supplementary S2: MATLAB algorithm for parameters estimation by non-linear regression

```
%nonlinear Fit for experimental data by Said Arévalo-Alquichire et. al. 2020
clc
%Creating matrix of coefficients for Mooney-Rivlin model
C=rand(3,1);
%Data reading from imported tables
data=out;
%Reading Engineering stress
stress=data(:,2);
%Reading engineering strain
strain=data(:,1);
%Transforming engineering strain to true strain
ts=1+(strain/100);
%transforming engineering stress to true stress
tss=stress.*ts;
%Defining parameter limitis for nonlinear regression
%upper bound,ub, and lower bound ,lb, limits for parameters regression
ub=[1e8,1e8,1e8];
lb=[1e-10,1e-10,1e-10];
options=optimset('disp','iter','LargeScale','off','TolFun',1e-5,'MaxIter',400,'MaxFunEvals',400);
%Non-linear regression syntaxis
[parameters,resnorm,residual,exitflag,output,lambda,jacobian]=
lsqcurvefit(@Mooneyrivlin,C,ts,tss,lb,ub,options);
%calculating model output
mod_out=Mooneyrivlin(parameters,ts);
%creating a matrix with model outputs
outputexp=[ts tss mod_out];
coeff=[parameters];
%exporting matrix outputexp to a txt file
name="PU678H.txt";
dlmwrite(name,outputexp,'delimiter','\t','newline','pc');
namecoeff="coeff"+name;
dlmwrite(namecoeff,coeff);
%Calculating Lin's concordance correlation coefficient according with Robert Matthew (2020). f_CCC
https://www.github.com/robertpetermatthew/f_CCC), GitHub. Retrieved April 7, 2020.
dataCCC=[tss mod_out];
alpha=0.05;
f_CCC(dataCCC,alpha);

function S = Mooneyrivlin(C,ts)
%Mooney-rivlin 3 parameters
S=((2.*C(1).*(ts-(1./(ts))))+(2.*C(2).*(1-(1./(ts.^3))))+(6.*C(3).*((ts.^2)-ts-1+(1./(ts.^2))+(1./(ts.^3))-
(1./(ts.^4)))));
```

```
End

function CCC = f_CCC(data,alpha)
% Computes Lin's Concordance Correlation    Coefficients CCC,
% Based on the development by Lin1989 and corrections in 2000, and presentation
% by McBride2005
% Data is returned as presented in the form returned by the CCC function in the
% R package 'DescTools'
% Lawrence, I., and Kuei Lin. "A concordance correlation coefficient to evaluate
% reproducibility." Biometrics (1989): 255-268.
% McBride, G. B. "A proposal for strength-of-agreement criteria for Lin?
% concordance correlation coefficient." NIWA Client Report: HAM2005-062 (2005).
% Syntax:
% Input:
%     data is an k by m matrix of k targets by m raters
%     alpha is the alpha level for significance using the confidence intevals
%     rho0 is the hypothesised value of ICC (set to zero if unsure)
% Output:
%     CCC
%     Scale and location shifts
%     Bias Correction Factor (gauge of accuracy)
%     Pearson's Correlation Coeff (gauge of precision)
%     Confidence limits at the significance given in alpha
% Example: (data from McGraw Table 6, modified to have shifted scale value)
% data = [103,109;% 82,65;116,106;102,102;99,105;98,100;104,107;
%             62,85;97,101;107,110];
% alpha       = 0.05;
% CCC = C_ICC(data,alpha);
% Verification from R:
%          est       lwr.ci       upr.ci
% 1 0.729616 0.2406287 0.9232147
%
% $s.shift
% [1] 0.9256678        (=1/1.0803)
%
% $l.shift
% [1] 0.1460437        (sign flipped)
%
% $C.b
% [1] 0.9865349
%
% $blalt
%       mean delta
% 1   106.0      -6
```

```
% 2     73.5     17
% 3    111.0     10
% 4    102.0      0
% 5    102.0     -6
% 6     99.0     -2
% 7    105.5     -3
% 8     73.5    -23
% 9     99.0     -4
% 10 108.5     -3
% RPMatthew 20180412
Ybar        = mean(data);
S           = cov(data,1);
r           = (S(1,2))/sqrt((S(1,1))*(S(2,2)));        % Pearson's corrleation coeff (precision)
u           = (Ybar(1)-Ybar(2))/(sqrt(sqrt(S(1,1))*sqrt(S(2,2))));   % locShift
v           = sqrt(S(1,1))/sqrt(S(2,2));                              % scaleShift
Cb          = ((v+1/v+u^2)/(2))^-1;                    % Bias Correction Factor (accuracy)
% rho       = (2*S(1,2))/(S(1,1)+S(2,2)+(Ybar(1)-Ybar(2))^2)
rho      = r*Cb;
Z        = atanh(rho);
E        = sqrt((...
    ((1-r^2)*rho^2)/((1-rho^2)*r^2)...
    +(2*rho^3*u^2*(1-rho))/(r*(1-rho^2)^2)...
    -(rho^4*u^4)/(2*r^2*(1-rho^2)^2))...
    /(size(data,1)-2));
z = @(p) -sqrt(2) * erfcinv(p*2);
clear CCC
CCC{1}.name    = 'Lin''s Concordance Correlation Coefficient';
CCC{1}.est                = rho;
CCC{1}.scaleShift         = v;
CCC{1}.locationShift      = u;
CCC{1}.biasCorrection     = Cb;
CCC{1}.pearsonCorrCoeff = r;
CCC{1}.confInterval       = [tanh(Z+z(0.5*alpha)*E),tanh(Z+z(1-0.5*alpha)*E)];
end
```

Supplementary S3: MATLAB algorithm for modelling of polyurethanes behavior on simulated physiological conditions

```
% Modelling of polyurethanes behavior on simulated physiological conditions.
clc
%Parameters
%frequency
w=1;%Hz
%Initial radius
r0=0.28/100; %m
```

```
r=r0;
h=0.38/100; %m
%presure range
pmax=180; %maximun pressure in mmHg
pmin=40; %minimum pressure in mmHg
%mean pressure
pm=((1/3)*pmax)+((2/3)*pmin);
%amplitud of sinosoidal pressure
ps=10; %mmHg
e=ps/pm;
%Initial value for ts calculation from hyperelastic model
ts0=1;
%Loop for solution of equations as function of time
for i=0:1000
t=1+(0.1*i);
%creating vector time
time(i+1,1)=t;
%Sinusoidal pressure
pmmhg=pm*(1+(e*sin(w*t))) ; %pressure in mmHg
p=pmmhg*133.32/1e6; %units conversion to MPa
%creating vector with pressure
%In Mpa
pr(i+1,1)=p;
%In mmHg
prmmhg(i+1,1)=pmmhg;
%circumferential stress calculation
s=(p*(r0^2)/(((r0+h)^2)+(r0^2)))*(1+((r0+h)^2/r0^2));
%creating vector with circumferential stress
stressc(i+1,1)=s;
%Stretch from hyperelastic model
y=fsolve(@(ts)dmr(ts,C,s),ts0);
%radius as function of time
r=(r0*(y-1))+ r0;
%creating vector with radius
radius(i+1,1)=r;
end
%calculating compliance and creating a vector
for j=1:1000

complaince(j,1)=(radius(j+1,1)-radius(j,1))/(radius(j,1)*(prmmhg(j+1,1)-prmmhg(j,1)));

end
```

```
function F = dmr(ts,C,s)
%Mooney-rivlin 3 parameters
F=-s+((2.*C(1).*(ts-(1./(ts))))+(2.*C(2).*(1-(1./(ts.^3))))+(6.*C(3).*((ts.^2)-ts-1+(1./(ts.^2))+(1./(ts.^3))-
(1./(ts.^4)))));
end
```

© 2020 by the authors. Submitted for possible open access publication under the terms and conditions of the Creative Commons Attribution (CC BY) license (http://creativecommons.org/licenses/by/4.0/).
